# Multi-Level, Multi-Component Approaches to Community Based Interventions for Healthy Living—A Three Case Comparison

**DOI:** 10.3390/ijerph13101023

**Published:** 2016-10-20

**Authors:** Bent Egberg Mikkelsen, Rachel Novotny, Joel Gittelsohn

**Affiliations:** 1 Department of Clinical Medicine, Aalborg University, Frederikskaj 10, Building: B, Room: B2, Copenhagen SV 2450, Denmark; 2 College of Tropical Agriculture and Human Resources, University of Hawaii at Manoa, Honolulu, HI 96822, USA; novotny@hawaii.edu; 3 Center for Human Nutrition, Johns Hopkins Bloomberg School of Public Health, 615 N. Wolfe Street, Room W2041, Baltimore, MD 21205, USA; jgittel1@jhu.edu

**Keywords:** Community Health Programs, multi-level interventions, multi component interventions, healthy living, Health and Local Community (SoL-program), Children’s Healthy Living (CHL), B’More Healthy Communities for Kids (BHCK)

## Abstract

There is increasing interest in integrated and coordinated programs that intervene in multiple community settings/institutions at the same time and involve policy and system changes. The purpose of the paper is to analyse three comparable cases of Multi Level, Multi Component intervention programs (ML-MC) from across the world in order to give recommendations for research, policy and practice in this field. Through the comparison of three cases: Health and Local Community (SoL-program), Children’s Healthy Living (CHL) and B’More Healthy Communities for Kids (BHCK), this paper examines the potential of ML-MC community-based public health nutrition interventions to create sustainable change. The paper proposes methodology, guidelines and directions for future research through analysis and examination strengths and weaknesses in the programs. Similarities are that they engage and commit local stakeholders in a structured approach to integrate intervention components in order to create dose and intensity. In that way, they all make provisions for post intervention impact sustainability. All programs target the child and family members’ knowledge, attitudes, behavior, the policy level, and the environmental level. The study illustrates the diversity in communities as well as diversity in terms of which and how sites and settings such as schools, kindergartens, community groups and grocery stores became involved in the programs. Programs are also different in terms of involvement of media stakeholders. The comparison of the three cases suggests that there is a need to build collaboration and partnerships from the beginning, plan for sufficient intensity/dose, emphasize/create consistency across levels and components of the intervention, build synchronization across levels, and plan for sustainability.

## 1. Introduction

Individual-level approaches to food and lifestyle behavior change, including through social marketing campaigns and education, have been largely unsuccessful in changing behavior at the population level [1]. Consequently, there is an increasing focus on interventions that focus on changes at the environmental, system and policy levels, particularly changes to institutions within community settings. Significant effort has gone into interventions in key community institutions, such as schools [2,3,4], pre-schools [5,6], worksites [7,8] and food stores [9,10], with some tangible results. Studies using “the settings approach” as defined in the Ottawa charter [11] have been able to show effects on food acquisition and consumption [12,13], as well as on physical activity [14]. However, interventions in single settings/institutions tend to rely on intensive short-term activities, and may have limited sustainability.

There is increasing interest in integrated and coordinated programs that intervene in multiple community settings/institutions (the community environment) at the same time [15,16,17,18,19,20,21,22] and involve policy and systems changes. Such programs tend to target multiple settings such as schools, parks, health clinics, supermarkets, corner-stores, restaurants, and worksites simultaneously—and do so in a coordinated manner to create greater intensity, effect, and sustainable systems change. However, interventions that act at multiple levels and use multiple components (e.g., education, policy, social marketing, and/or advocacy) are challenging in terms of development, effective implementation evaluation, cost, and management [23,24].

Multilevel-Multicomponent (ML-MC) interventions are interventions that work on more levels at the same time and that involves more intervention components that acre synchronized across levels. As a result, they require extensive community engagement. However, applying participatory research principles/co-creation in community interventions involves balancing the need for the production of scientific evidence with the need to be locally and socially acceptable [25]. Attention to community needs, wants and strengths is required for effectiveness and sustainability [25,26]. Synchronization and coordination of intervention action across different settings requires managing conflicting agendas and priorities among the settings and its stakeholders. Creating and measuring the necessary intensity and “dose” is a challenge, as is choosing the appropriate evaluation methods and study designs that are able to measure the effect of complex interventions.

The aim of the paper is to compare the three programs, to outline differences and commonalities, strengths and weaknesses, and to provide recommendations for future programs/intervention trials. It does so by comparing three ongoing cases of such interventions from around the world. The paper discusses similarities and differences in approaches and examines the strengths and weaknesses in these ML-MC interventions. The paper aims to identify and propose methodology, guidelines, and directions for future research.

## 2. Materials and Methods

The three cases of Multilevel-Multicomponent (ML-MC) community based behavior change interventions were:

Case 1: Promoting healthy eating and non-sedentary behavior in the Local Community SoL-program {from the Danish (Sundhed og Lokalsamfund)—Health & Local Community} [27,28].
Case 2: Environmentally focused community randomized intervention trial for young child obesity prevention: Children’s Healthy Living (CHL) for Remote Underserved Minority Populations of the Pacific [29].
Case 3: B’More Healthy Communities for Kids (BHCK): a multi-level obesity prevention program for low income urban African American children [30].

The three cases were presented as papers and made up a session on community-based interventions held at the annual conference of the International Society on Behavioral Nutrition and Physical Activity [31]. The presentations, as well as the final Q + A session and discussion after the presentations, served to identify key questions to be addressed by the authors from each of the three individual programs, across several domains. The creation of comparison tables was completed by the lead investigator of each project, and subsequently seen by the assistant program director/project manager for each study.

The format of the comparison tables was developed by the three coauthors, and then completed by the respective co-workers involved in each of the three programs. Successive rounds of review and comments by the coauthors were then conducted to refine and enhance comparability of the tables.

Ethical approval: all subjects gave their informed consent for inclusion before they participated in the study. In the case of children, permission was given by caregivers. The studies were conducted in accordance with the Declaration of Helsinki, and the protocol was approved by the Ethics Committee in each of the three countries.

The SoL—Health & Local Community program was approved by the Institutional Review Board, the Ethical Committee of the Capital Region with the Project Identification Code: 3-2013-036.
The Children’s Healthy Living program was approved by the University of Hawaii at Manoa Committee on Human Studies number 18915.
The B’More Healthy Communities for Kids program was approved by the Johns Hopkins School of Public Health Institutional Review Board. IRB number 00004203.

The common denominator for the three programs was that they were all integrated and coordinated programs that intervene in multiple community settings/institutions across the community environment in a synchronized manner and at the same time. However, the programs differed slightly with respect to the nature of the intervention components due to differences in context and cultural traditions. Table 1 gives an overview of the intervention components of the three programs.

All three programs were monitored by detailed formative or process evaluation. For the SoL—Health & Local Community program the formative studies focused on measuring children’s perspectives on health, examination of consumer practices and perceptions in relation to food store shopping, examination of food retailer perspectives on their role and responsibility in relation to promoting healthy food choices, investigation of the role of the mass media stakeholders including the local television, radio and newspapers in health promotion, examination of citizens program awareness and media habits and investigation of the motivations and barriers of community-based stakeholders and citizens to contribute to engage in health promotion action in the local community.

For the Children’s Healthy Living program, monthly implementation reports were completed for each community participating in the intervention. This was done by the project staff of the program. The report acceptability, reach, likelihood of effectiveness, adoption, sustainability, and feasibility scoring according to set specific criteria were recorded.

The B’More Healthy Communities for Kids program conducted a detailed process evaluation at multiple levels of the intervention (policy, wholesaler, cornerstore, carryout, recreation center, peer leaders and social media). The program staff measured reach—percentage of the target population who received any component of the intervention, dose delivered—amount of each intervention provided by program staff as well as fidelity—level of engagement with project activities by the target population. All process measures were assessed according to set standards, and characterized as low, medium or high. Bimonthly meetings of the project team were conducted to assess implementation quality and to improve the intervention in future stages.

## 3. Results

Table 2 compares the study designs of the three Multilevel-Multicomponent (ML-MC) interventions, including setting, target population and forms of engagement with local communities. In Table 3, we characterized the three programs with respect to how they were evaluated. All three programs have strong and ambitious evaluations underway. Evaluation strategies target the effectiveness of the intervention to change the behavior and environment at multiple levels using a broad range of outcome measures. All programs include process measures describing and quantifying how the intervention was implemented and providing insight into the process of change. All programs include behavioral outcomes as well as indicators of health status at the individual level as well as measures at the institutional level (stores, parks, recreation centers). The Children’s Healthy Living program and B’More Healthy Communities for Kids also evaluated the intervention at the community/neighborhood level. The sample sizes ranged from 1448 to 4443 individuals, involving 200 to 724 families. The number of targeted communities/neighborhoods ranged from 3 to 30.

### 3.1. Similarities

Comparison of the three ML-MC intervention trials show both diversity as well as commonalities (Table 3). The programs similarly worked to build the intervention with the community. All three deliver multiple intervention components at the same time, and all are doing so across multiple levels and settings in a coordinated manner aiming at enhancing intervention intensity and effect.

All three programs have actively engaged local stakeholders in the development and implementation of intervention components while maintaining a strong research design. The SoL/Health & Local Community and Children’s Healthy Living programs are similar in the way that they are implemented in island settings, adding a dimension of remoteness—and in remote Alaska in the case of the Children’s Healthy Living program. The fact that intervention environment is made up of islands has implications both for evaluation and for intervention delivery, since it can be assumed that interventions can be delivered in a more intense manner due to the limited mobility of islanders compared to individuals living on the mainland.

All three programs are using several strategies to target the child and family members’ knowledge, attitudes and behavior through education, awareness-raising and information provision, as well as by modifying the food and physical activity environment through structural changes—such as creating new options in community food and physical activity environments. All three programs are seeking to sustain their impact post intervention by working with many stakeholders through policy changes and coalition building while at the same time carrying out their evaluation and other research tasks.

All three ML-MC programs have a primary focus on prevention of chronic disease through changes in food and physical activity behavior. All intervene at multiple levels and multiple settings, and engage a broad range of stakeholders to create, deliver, mediate and facilitate the intervention components with the target group. They all use the community or neighborhood as the primary unit of intervention and evaluation. They all have a strong family component as caregivers and relatives are important facilitators in the microsphere of young people. The duration of the intervention programs range from 10 to 38 months.

### 3.2. Differences

The three ML-MC interventions differ substantially in terms of the specific community institutions in which they were implemented. The differences might be explained through cultural, national and historical differences in the communities, age of the targeted youth, as well as through different disciplinary traditions in both the research and policy systems engaged in the programs. The programs also differ in terms of how specific sites and settings of the local communities are involved in the programs. For instance, in the case of SoL/Health & Local Community, the part of the program addressing schools and kindergartens, the school and kindergarten teachers play an important role in facilitating the intervention. The Danish school system is currently undergoing significant reforms including the introduction of longer school days that calls for a rethinking of the way that food provision is handled. This has led to a new type of “foodscape” thinking in which food is not only thought of as meals to be served but also as an opportunity for learning about food, nutrition and health based on the insights from the Whole School Approach [32]. The food environment in Danish schools is diverse since there are no national policies setting out rules for the provision. Instead, school foodscapes [33,34] are designed through bottom–up approaches where local agency and commitment are driving the development of different ways of providing food. The options for influencing and intervening in the school foodscapes range from implementing school fruit schemes, school milk schemes, breakfast clubs and lunch arrangements. Food and meals in these cases could be attached to the classroom or to a central cash cafeteria. In addition, food supply interventions can target the in-class room curricular activities. Thus, in the case of SoL/Health & Local Community, there has been a broad range of food activities that could be targeted.

In the case of B’More Healthy Communities for Kids, schools are not a focus. This choice is based on the constraint that teachers did not have the time or the resources to take on additional curricula and programs—and also on the very low access to healthy food choices in the community environment. The researchers felt it a priority to enhance access to healthy foods in this setting where very few such options are available. It is not enough to work with just the direct sellers of foods in these urban communities, but also with those wholesalers and distributors who supply the foods.

In the case of the Children’s Healthy Living program, among indigenous populations, there is high value, motivation and broad interest in reinvigorating cultural, traditional locally grown foods. With the Children’s Healthy Living program’s focus on indigenous populations who have experienced a nutrition transition to western foods, which is associated with chronic disease, there is strong motivation to strengthen indigenous food systems throughout the community’s institutions, and support cultural practices that support health.

The three case studies are clearly different in how they address food shopping behavior. The format of retail food stores is significantly different in the three cases. In the SoL/Health & Local Community case, food shopping is based on small supermarkets and groceries set in small villages with only few food retail options. In Baltimore, the low income population has ready access to small corner stores and carry-outs which have limited availability of healthy foods, but very few supermarkets. In the case of the Children’s Healthy Living program, the native population is motivated to reinforce cultural food patterns, which provides opportunity to reinforce growing and eating native/local fruits and vegetables, in addition to enhancing fresh local foods in the community food stores, which vary in size in the communities. Currently retail foods are largely imported.

The programs are also different in their approaches to involving the media actively in their intervention efforts. In the case of SoL/Health & Local Community, the local TV network plays an important part of the intervention in raising awareness of the intervention. As such, the delivery of communication is based on a long-term strategic agreement between the program management and the media partner. The SoL/Health & Local Community program also involves use of social media (Facebook). For the B’More Healthy Communities for Kids program in Baltimore, an emphasis has been placed on social media (Facebook, Twitter, and Instagram) and text messaging a means of reaching the target population. In the Children’s Healthy Living program, social marketing was limited to the community level, during the intervention, to minimize bleed of this information into the control communities that were geographically close by. Community level social marketing included development and distribution of information sheets, placemats, and a development of a native superhero card series that promoted each of target behaviors. Some text messaging was done in Alaska which had infrastructure for targeted messaging.

### 3.3. Key Components

All three programs were faced with a number of important common challenges. Importantly, they all developed similar strategies for dealing with those challenges, providing guidance for future work.

The comparative analysis identified five challenge areas: (1) building collaboration and partnerships; (2) creating intensity, dose and effectiveness of intervention activities; (3) creating consistency between activities and across levels; (4) synchronizing program activities across institutional settings and levels; and (5) designing the intervention programs to be sustainable post intervention. In Table 4, an overview is given of how the three programs are dealing with the key challenges of ML-MC community-based intervention trials.

#### 3.3.1. Need to Build Collaboration and Partnerships from the Beginning

Experience from the case studies shows that community partnership, from the beginning, is crucial for ML-MC programs. Community partnership creates the foundation for development of the intervention program—and its potential for sustainability. The researchers must engage in this work before, during and after seeking funding, identifying important stakeholders, leaders and others active in this work and identifying significant action possibilities in a given local community at a given point in time. This is accomplished by convening stakeholders and developing and maintaining community relationships and convening and building vision, mission, goals and prioritizing while considering do-ability among those with vested interest in the work, who already in some way do some aspect of the intervention, and who will be responsible for the delivery of the intervention activity in some way. The management and development of relationships is time consuming, usually requiring long-standing relationships and a record/reputation of good partnership with the community. It requires close attention from the researchers on issues of how to evaluate the program and a realistic and efficient research protocol that yields results, requiring creating and presenting a viable process and plan to potential funders and acknowledging limitations, in order to manage expectations. The importance of the planning process should not be underestimated in terms of level of detail and amount of time needed. Involving key stakeholders in the planning and implementation of the intervention is essential for investment in the program, and is required for long-term program success and sustainability.

Preliminary studies and piloting can be ways of testing components on a small scale, for feasibility and for stakeholder acceptance, as well as for user acceptance and compliance. In the case of SoL/Health & Local Community, the participatory planning process focused on identifying key stakeholders in a series of participatory kick-off meetings. These became organized as a loosely coupled island-wide partnership. At the village level, local community action groups were formed (Community Action Groups—CAGs). Key leaders/role models in each community and jurisdiction were identified and invited to suggest action and prioritize options [25,35]. In the case of B’More Healthy Communities for Kids and Children’s Healthy Living, local agencies were hired to develop and implement aspects of the intervention to assure local experience and acceptability of the intervention. In these cases, individuals and agencies were selected who already did similar work, a positive deviance approach-building on what is already working [36]. The Children’s Healthy Living program was possible due to earlier development of a region-wide coalition (Healthy Living in the Pacific Islands) over about 15 years of partnership [37] and from prior work (Healthy Pacific Child Project, including Healthy Foods Hawaii) which built partnership and experience to develop component activities [38]. In B’More Healthy Communities for Kids, a pilot trial of an urban farms to cornerstore program led to the decision to not involve these farms in the program [39]. The community engagement process in the case of B’More Healthy Communities for Kids was conducted through policy working groups, sequential workshops, and multiple trainings of community implementers (e.g., small store owners, youth leaders, etc.) that were designed to provide adaptation and sustainability of the intervention components [13].

#### 3.3.2. Plan for Sufficient Intensity/Dose

Intensity is about creating synergy and impact by ensuring that different components of the intervention reinforce each other, and by creating repetition of program activities and messages. The aim of creating intensity is to create action and compliance with the intended behaviors in the target group by using a multiple-exposure approach. This, in many cases, requires compliance not only by the target group but also by the key stakeholders and facilitators that are intended to deliver the key components and key messages about behavior change. In the case of SoL/Health & Local Community program, intensity was addressed through giving high priority to the development and management of the relationships with key stakeholders and facilitators responsible for the intervention delivery. In addition, the local stakeholder groups were used to plan the timing and content of the different intervention components. Through these actions, compliance was strengthened while at the same time developing and reinforcing the relationship with the CAGs and their members, which, in many cases, were also facilitators of the intervention at the same time. In the Children’s Healthy Living program, community role models were identified who already practiced desired behaviors, who were supported to role model even more. Monthly progress reports were made on what, where, who and how many of each type of activities occurred with which subpopulations to reach each of the target behaviors. Coalition meetings allocated work to those groups whose mission most closely aligned with the activity to create efficiency, coordination and sustainability. In the case of B’More Healthy Communities for Kids, reinforcement was created by having each intervention level linked to other levels in order to emphasize specific promoted foods, behaviors and messages. B’More Healthy Communities for Kids identified standards for delivery of each intervention component with the aim of assuring sufficient intensity of intervention delivery, and conducting reviews of implementation success and failures every two months, with appropriate revisions to implementation of the program as needed [13].

Creating intensity by engaging local champions in the interventions was a key strategy applied in all three cases. In the Children’s Healthy Living and SoL/Health & Local Community cases, the strategy included looking after (including hiring them, and providing scholarships to relevant students) the persons at a local level, who can actually make a difference and act as an ambassador for the intervention ideas and intervention. Involving these champions in local partnerships can also be a way to address long-term sustainability of the program. Partnership approaches contribute to creation of more local ownership and possibilities to anchor programs permanently. For B’More Healthy Communities for Kids, a key strategy was training a cadre of 16 youth leaders who directly engaged with young people at recreation centers and in other community venues. Children’s Healthy Living also engaged youth role models who led activities with younger children. The cases show that the intervention program, to a large extent, rests on the combined efforts of the researchers and local community stakeholders, and to local capacity building.

#### 3.3.3. Emphasize/Create Consistency across Levels and Components of the Intervention

Consistency and synergy must be created between different intervention component activities. Different components of the ML-MC intervention should emphasize the same things, using the same language and the same framing of health messages across communities with a similar study design assignment, and across jurisdictions (in the case of Children’s Healthy Living). In many cases, this was done through integrating the health messages and actions into activities already taking place in the local community, in particular, for the communication of intervention messages through media channels. The last point is crucial. In the case of SoL/Health & Local Community, the program consistency was accomplished through a participatory action research approach involving frequent visits to assure compliance with protocol and, for each intervention component, a Standard Operational Procedure (SOP) was developed. A strategy for communicating health messages was developed and the thematic framing was discussed with the media partners. In many cases, these messages have been integrated with traditional activities and events already taking place on the island. The Children’s Healthy Living program developed a template that was used by each jurisdiction to develop and track intervention components and used quality assurance visits to assure consistency while assisting in developing appropriate modifications and adjustments for a particular setting. In the case of B’More Healthy Communities for Kids, the importance of meeting a series of minimum delivery standards for each program component was emphasized through training and through an Interventionist Manual of Procedures. Consistency means that delivery of each intervention component happens in a similar way from setting to setting.

#### 3.3.4. Build Synchronization across Levels

Synchronization is about making sure that the intervention components and activities that are taking place at the different levels are synchronized in terms of optimal timing and involves creating a sense of coherence in the themed intervention activities. Activities that are presented in later stages should build on those provided in earlier stages. The key requirement for synchronization is a well-recognized aspect of evidence based knowledge and flexibility; it reflects openness to engage in co-creational processes with local stakeholders. As such, the final decisions on when to perform different activities, as well as decisions on what to do, is a negotiating process in which researchers provide inspiration, evidence and ideas and in which local stakeholders provide “local evidence” on “what might work” and when would be the best time to launch the activity in question.

Timing and sequence of activities is the product of active community partnerships. In the case of SoL/Health & Local Community, the guiding principle for the building of synchronization was that families would be experiencing the same messages in the mediascape, while at the same time learning about the same health messages and themes when their children come home from school and encountering the same theme when doing the daily shopping. The SoL programs’ health messages were provided through the TV network and through local intervention facilitators as well as through provision of ideas using a catalogue of ideas that should give local stakeholder inspiration and support the synchronization of the ideas across settings. In the case of Children’s Healthy Living, this topic was addressed through a provision of a template of activities and regular conference calls and visits. In the Children’s Healthy Living Program, a template of the intervention components was created based on a review of the evidence-based literature and a process of community input [25]. Activities were sequenced, for example, gardening began early, so that food from the garden could be showcased, modeled and used for examples at community gatherings in later stages of the intervention. The stage of behavior change theory was applied to the community activities where early activities provided information, later activities adapted to local circumstances, and the last activities were designed to sustain, maintain and transfer the program to community leadership. The B’More Healthy Communities for Kids intervention was conducted in a series of phases, with specific targeted foods, messages and behaviors for each phase. The intervention team negotiated between intervention levels (e.g., wholesalers, small stores owners, carryout owners, youth leader training, social media coverage) to ensure timing and readiness for each component (e.g., cornerstores are stocking the foods promoted through social media and at recreation centers by youth leaders).

#### 3.3.5. Plan for Sustainability

Sustainability requires creating the foundation for the program and intervention activities to continue after the research team has ceased most or all activities. An important approach identified in all programs is the need for capacity building of those who will be responsible for implementing the intervention in the future. Such training and education can target nurses, school and kindergarten teachers, pre-school teachers, health care staff and staff in local community clinics. Training and education could take place as in-service training and also needs to be built into the existing higher educational system at under graduate and graduate levels.

In the case of the SoL/Health & Local Community program, the need for capacity building has been addressed by collaborating with the local educational programs for nurses, school and kindergarten and by planning for a training of program ambassadors among the local workforce. This approach is aiming at handing over the responsibility of the future SoL/Health & Local Community program to the local leaders while the research team at the same time is stepping back. In the case of Children’s Healthy Living, this is addressed by supporting community role models, by providing scholarships for degree training for each jurisdiction, and by building coalitions of community partners [22] aimed at carrying forth this work. Sustainability is also occurring in the university by integrating the learning outcomes and insights from Children’s Healthy Living into university curricula, and by offering more adult education and community education using distance training, and collaborating with regional agencies, including the World Health Organization (WHO). In the case of the B’More Healthy Communities for Kids, the researchers developed a series of capacity-building trainings, including narrated power-point presentations to make information on program compliance available to store and carryout owners, and by developing a 15-session training session for youth leaders and involving them in program development and maintenance. All cases seem to have good experiences with applying strategies for engaging stakeholders from multiple institutions in order to build ownership and sustainability. Children’s Healthy Living Program highlights the added value of providing salary/training to community workers/agencies, providing degree training to future professionals from the region, and using data for policy advocacy. B’More Healthy Communities for Kids addressed the topic through the establishment of policy working groups tasked with sustainability. In addition, as many of the materials developed as possible (including training materials) were made available online.

## 4. Discussion

This is one of the first papers to compare ML-MC intervention programs. Our analysis shows that using the full range of approaches in health promotion is important. All of the following three strategies are key to a successful implementation of ML-MC interventions: education/information, environmental change and policy change, and, as a result, they should be a part of all components of the ML-MC intervention. The comparison of the three cases has identified five key actions that in all programs have been found to be key to successful community intervention implementation. They are: building collaboration, creating intensity, ensuring consistency, assuring synchronization and creating sustainability of the intervention. The comparison of the programs points to some of the methodologies and approaches that the programs have applied in order to address these challenges.

### 4.1. Policy

The three ML-MC programs are all advocating for and working on local community action at the policy level. In the case of the SoL/Health & Local Community program, relationships with policy makershave drawn in the insights and evidence from the National health surveys that provide data on the health outcomes and behaviors of the population, including that of the intervention site at the isle of Bornholm. Such evidence plays an important role in the decision making process in the local municipal government. In the later stage, the activity at the policy level involves partly handing over the responsibility of the post intervention SoL/Health & Local Community 2 program to the community and Municipality. This includes negotiations and discussions on how to organize the future activity, how to finance it, and how to establish a governance structure and share the responsibility between municipal actors and civil society and market actors in a sustained partnership. Children’s Healthy Living is working to leverage the partnerships and the data being collected on this underserved population to advocate for policy change. In fact, the ability to provide ongoing data for policy action has emerged as a priority. One way to address the need for providing the decision tools to policy makers is the use of the simulation models—as are being used for B’More Healthy Communities for Kids 13. An agent based model of food foraging behavior of low income children in Baltimore was developed by engineers, nutritionists, public health researchers and anthropologists and is able to answer “what if” type of questions and can be used to assist in making informed decisions on intervention components based on a forecast of the potential outcomes such interventions could have.

### 4.2. Implementation

The comparison also illuminates a common emphasis on assessing implementation successes and challenges. All three programs put a strong emphasis on assessing process measures. Since interventions at multiple levels and with multiple components often are very time-consuming to develop and since the lag time is often considerable, such programs are often forced to give in to the possibility of demonstrating quick and clear effects on traditional outcome measures. Programs are in all cases faced with the challenge of how to be able to demonstrate both clear effects on traditional outcome measures in a well-defined sample of participants and at the same time be open to participatory approaches and co-creation with local stakeholders. A general lesson learned across cases suggests that program evaluator’s focus on overall effects. It is also important to develop methods for ongoing monitoring and evaluation that are suitable and acceptable in terms of scientific standards and at the same time low-cost and easy to administer for the local stakeholders to assess and use after researchers have left the program.

The paper adds to the growing literature on community based interventions (CBIs) and programs (CBPs) addressing unhealthy eating and sedentary behavior in various settings in the local community and with community leaders and workers as facilitators and co-constructors of the interventions. One challenge here is the considerable lag time that occurs from conception, initiation, and implementation to analysis and publishing of results. One of the programs with a sufficient history is the Shape Up Somerville (SUS) program—a large community-based environmental intervention which results has been reported in a number of papers [17,40,41,42,43]. The program dates back 15 years and was initiated in 2002 as a Centers for Disease Control-funded research study led by Somerville community members and researchers in the state of Massachusetts. The program aimed initially at preventing obesity among student 1st to 3rd grade but broadened its reach and scope later on. The program has been faced with some of the same challenges as the three ongoing programs reported in this paper. The program applied a multi-level strategy addressing policy and environmental changes at the same time as applying educational strategies such as posters, table tents, menu boards, nutrition information, etc. to promote healthy eating as well as strategies to reach at-risk populations such as ethnic minorities, immigrants, and low-income citizens. In the same way as the three presented programs, the SUS intervention has been taking a broader approach addressing wider determinants of food choice as the program developed. Interventions and activities have involved items such as farmers’ markets, school gardens and farm-school linkages [44] and improvements in school food service, as well as elements of urban agriculture.

Viewing the 15-year history of the SUS program provides insight into the long-term fate of complex community based health programs. Like our studies, SUS was initiated by researchers in a close cooperation with community opinion and community leaders. Following the three-year study, the program was adopted by the City of Somerville and has continued to evolve as a community wide and participatory approach from which other local community programs have drawn inspiration. As such, it provides valuable insight in the dynamics related to the transformation from a research program to a sustained and institutionalized phenomena. The Shape Up program shares characteristics with SoL/Health & Local Community program and BHCK in the way that it has developed as a primary food and eating focused intervention that has gradually come to involve physical activity as well. This has included components such as community path developments, green line subway extension, and physical activity programs at the school day, community walkability and bikeability interventions.

Evolving from initially only intervening in school settings, the program has developed to spread over more settings to include worksites and restaurants, and, as such, it corresponds well with the insights from the reported programs. It also corresponds well in the way that it has been prioritizing focus on already ongoing activities and been considering how connections and links can be made to what the community already values and engages in, as is done with coalition building in Children’s Healthy Living. Attaching to already existing activities might provide the opportunity to change them into more effective interventions providing more “health for money” and at the same time creating synergy between what is socially accepted and what is effective.

The comparison of the three multi-level, multi-component approaches promote healthier living through community based interventions and demonstrates that this area of research is of high relevance. Around the world, a new type of governance seems to be emerging in which researchers engage with a broad range of stakeholders in the local communities. The belief in the potential of local actions as alternative to actions at the national level seems to be growing as is the belief in the fact that no single actor in society can solve problems on unhealthy eating and lifestyles. The comparison points to a number of challenges that need to addressed in future research and that has been identified in our study. The need for building collaboration and partnerships with a broad range of stakeholders and opinion leaders in the community is important and is important at an early stage of the program development. The creation of impact through the development of actions at more levels and through more components is important in order to make sufficient intensity and dose of actions. In addition, these actions need to be further developed and implemented in a coordinated, synchronized and consistent manner in order to be able to make sense to both facilitators and target audiences. Finally, a well planned strategy for measures that can enhance for sustainability of intervention through the involvement and alliance-building between partners is considered as very important.

It should be noted that the limitations of this work are that programs are not yet complete, and, as a result, the impact on a longer term is not known. It is also important to note that the political, economical and cultural contexts of the different programs analyzed are very different. Finally, it should be kept in mind that the development of local community programs is dependent on funding, especially if they are to be evaluated using reliable methods. Different worldwide approaches to the funding of projects have been explored, but there is a need for increased cooperation and exchange of experiences on that topic.

## 5. Conclusions

ML-MC interventions are complex and require a depth of experience to conduct. They require building collaboration and partnerships from the beginning, planning for sufficient intensity/dose, emphasis/creation of consistency across levels and components of the intervention, building synchronization across levels, and planning for sustainability.

## Figures and Tables

**Table 1:** Multilevel-Multicomponent (ML-MC) intervention components. The table shows the components of the three ML-MC community-based interventions.

Setting/Component	SoL/Health & Local Community	Children’s Healthy Living	B’More Healthy Communities for Kids (BHCK)
School/Kindergarten/Preschool	Taste workshops, gardening, lunch box workshops, food store educational tours, fish and fruit and vegetable eating promotion	Preschool Wellness Policy evaluation, Gardening, Role Model Training, SPARK physical activity training for teachers (Sports, Play, and Active Recreation for Kids)	Not applicable in this program
Family	Gardening, flyers, Social Media	Gardening, Heroes for Health cards	Not applicable in this program
Food stores/corner stores/Road side stands	Taste workshops, price reductions, interior design, space management, décor adjustments and choice architectures	Food cost assessment, Healthy food assessment, healthy food makeover	Increased access to healthy, affordable foods in corner stores and carryouts. Wholesalers stocked and promoted healthier foods. Point of purchase promotions in each venue (shelf labels, posters, interactive sessions).
Social marketing-TV networks/text/email/web	Just a little healthier TV series, strategic media partnership agreement, press releases, feature stories, Social Media groups	Email/Web/paper Newsletter, text reminders for activities, sandwich boards	Facebook and Instagram accounts targeted adults. Twitter account targeted city stakeholders. Text messaging program targeted adult caregivers of intervention youth.
Training in Obesity prevention skills	Not applicable in this program	Scholarships for University degrees for 21 selected citizens, Role model training, SPARK physical activity training	Trained 28 high school and college students to be youth mentors
Municipality/Community	Health policy provisions. Local action group establishment	Coalition development, Role model training, Community Leader readiness for change assessment	Policy working group brought together city council members, representatives from city health department, schools, recreation and parks, and other key stakeholders to plan policy initiatives and sustain program activities
Parks/Recreation Centers	Not applicable in this program	Playground building, sports equipment exchange	Recreation centers served as venues for mentor-youth interactions and training. Youth mentor-led nutrition/cooking lessons for adolescents (aged 10–14 years).

**Table 2:** Characteristics of the three Multilevel-Multicomponent (ML-MC) community-based intervention trials.

Characteristic	SoL/Health & Local Community	Children’s Healthy Living	B’More Healthy Communities for Kids
Primary aim(s)	To increase healthy eating and decrease sedentary behavior	To facilitate the development of and to support social/cultural, physical/built and political/economic environment to promote active play and intake of healthy food to prevent young child obesity	To increase affordability, availability, purchase, and consumption of healthy foods by low income AA children, and reduce obesity
Setting	3 villages, middle income, above average rates of overweight, high blood pressure	27 predominantly indigenous Pacific island and Alaska communities in 5 Pacific Jurisdictions (Alaska, American Samoa, Commonwealth of the Northern Mariana Islands, Guam, Hawaii)	30 low income, urban communities/neighborhoods in Baltimore City, MD, USA
Study Design	Community trial, baseline, follow-up 1 and 2	Community randomized trial	Neighborhood randomized controlled trial
Institutions involved in intervention	Supermarket/retail, schools/daycare and media (TV, Radio, Print media)	Preschools, stores, parks, Physical Activity facilities, Fastfood restaurants, community-based agencies	Recreation centers, corner stores, carryouts, wholesalers
Target population	Children aged 3–8 years and their families 1st target. Other islanders 2nd target	Indigenous 2–8 year-old children and their families, preschool and native communities	Low income African American children, aged 10–14 years, and their adult caregivers
Duration of intervention, months	24	24	8–10 months in 2 overlapping waves
Key stakeholders	Representatives from health, youth/school and culture/leisure. Elected and civil servant level. High level and local management level of retailers. Local school and daycare headmasters. Local TV station manager and other local media actors. The three academic partners.	Preschool teachers, school administrators, health center personnel, parents, community not-for-profit agencies, elected officials, store owners, park officials, community leaders, role models, local college/university faculty staff and students.	Policymakers, city agency staff, wholesale store managers, small store and carryout owners, recreation center directors and staff, youth leaders, low income families. School of public health faculty, staff and students.
Forms of engagement of policymakers and key stakeholders	Three local village based citizen actions groups (CAGs). One island wide loosely couple partnership alliance consisting of key stakeholders from market, public and civil society	Guided by local advisory committees, support community role models from different sectors, support and facilitate action by community coalitions, convene stakeholder groups, enhance work of preschools and other community groups working with young children; provide scholarships to college for 2 students from each Pacific jurisdiction	Policy working group, Use of systems science modeling for engagement, regular meetings with key stakeholder groups, trainings (in person and online) of food source owners and youth leaders, social media

**Table 3:** Evaluation strategies of the three Multilevel-Multicomponent (ML-MC) community-based intervention trials.

Characteristic	SoL/Health & Local Community	Children’s Healthy Living	B’More Healthy Communities for Kids
Levels evaluated	Supermarket, schools/daycare, child, family, citizen	Individual child and adult caregivers, community stores, community parks, physical activity facilities, fast food restaurants, preschool teachers and administrators, community leaders; Community; Pacific jurisdiction	Child, adult caregivers, youth leaders, small food source, recreation center, wholesaler, policy makers
Process measures	Action competency, program awareness, perceived barriers for compliance among citizens and mediators	What, where, how many and who participated in each intervention activity—aimed at each of 6 target behaviors; quality assessment of implementation of each intervention activity; post-intervention assessment of intervention exposure	Reach, dose, fidelity of implementation at each intervention level (SMS, social media, youth leader, small food source, recreation center, wholesaler, policy)
Psychosocial/socio-cultural measures	Knowledge and attitudes in families	Cultural affiliation, household characteristics and food security	Knowledge, self-efficacy, intentions, outcome expectations of youth (aged 10–14 years) and their adult caregivers
Behavioral measures	Dietary intake and sedentary behavior	Two-day food and activity logs, sleep questionnaire, screen time questionnaire, accelerometry	Youth diet (FFQ), food purchasing and preparation; adult food preparation and purchasing
Health outcomes	Anthropometric status Weight, height, skinfold measurements waist- and hip circumference	Child weight, height for BMI, waist circumference, acanthosis nigricans	Change in youth and adult caregiver weight and height (BMI)
Other measures	Retail sales and public procurement figures	Community food (thrifty food) and utility costs; community food and PA environment assessments (120 stores, 150 schools; 88 physical activity facilities; 119 fast food locations; 102 churches; 227 food stores; 203 food store environments; 48 walking environments), community readiness for change in leaders	Stocking and sales of promoted foods in participating food sources
Sample size	In 6 high intensity villages: 841 children enrolled from 12 schools and kindergarten (total in case and control) In the low intensity areas: 1500 in case and in control (3000 in total)	27 communities (9 intervention, 9 matched control, 9 temporal) in 5 jurisdictions; 4483 indicator child-caregiver households at baseline *	30 urban neighborhoods; 724 child-adult dyads (24/neighborhood); 1 recreation center/neighborhood; 3 cornerstores/carryouts/neighborhood

SMS: Social marketing scheme; FFQ: Food frequency questionnaire BMI: Body mass index; * Some households have more than one child.

**Table 4:** Multilevel-Multicomponent (ML-MC) Toolkit. Approaches for meeting the challenges of ML-MC community-based intervention trials.

Creation of	SoL/Health & Local Community	Children’s Healthy Living	B’More Healthy Communities for Kids
Collaboration and partnerships	Key stakeholders identified in a series of participatory kick-off meetings. These became organized in loosely coupled community wide partnerships. At village level local community groups were formed (CAGs)	Key leaders/role models identified in each community and jurisdiction (e.g., state, territory) for partnership; coalitions developed; scholarships provided for education in obesity prevention; policy work groups	Community engagement process (policy working group, sequential workshops, etc.) designed to provide adaptation and sustainability
Intensity	Relations management across intervention settings and neigbourhoods Based on a participatory action research approach	Monthly progress/activity reports utilizing the RE-AIM framework	Reinforcement by having each intervention level linked to other levels; Limit the number of promoted foods, behaviors, messages and repeat them throughout multiple components of the B’More Healthy Communities for Kids.
Consistency	Frequent meetings with stakeholders and visits to intervention settings to assure compliance with protocol	Quality assurance visit and weekly conference calls	Criteria for approval of each intervention component; Develop and uphold minimum delivery standards; Training of staff, youth leaders, food source owners and staff; develop Interventionist Manual of Procedures
Synchronization	Addressed through advance planning of activities and in cooperation with CAGs as well as with facilitators at intervention settings	Template of activities according to stage of change theory	Intervention in a series of phases, with specific targets; Intervention team negotiates between intervention levels to ensure timing and readiness
Sustainability	Development and maintenance of relations with CAGs and the community partnerships. Creation of municipality commitment and integration of SoL/Health & Local Community approach in municipal health strategy	Add value (salary/training) to community workers/agencies. Provide degree training. Policy advocacy for change with data. Community coalitions; adoption of activities by community partners; capacity building through training and role model development; improvements to the environment. Colleges as backbone organizations.	Policy working group tasked with sustainability; Trainings to enhance capacity-building

CAGs: Community Action Groups; RE-AIM: Reach Effectiveness Adoption Implementation Maintenance Framework.
